# Decision Tree for Early Detection of Cognitive Impairment by Community Pharmacists

**DOI:** 10.3389/fphar.2018.01232

**Published:** 2018-10-29

**Authors:** Maria Teresa Climent, Juan Pardo, Francisco Javier Muñoz-Almaraz, Maria Dolores Guerrero, Lucrecia Moreno

**Affiliations:** ^1^Community Pharmacist, Valencia, Spain; ^2^Embedded Systems and Artificial Intelligence Group, Universidad CEU Cardenal Herrera, Valencia, Spain; ^3^Department of Pharmacy, Universidad CEU Cardenal Herrera, Valencia, Spain

**Keywords:** memory complaint, early detection, mild cognitive impairment, sleep duration, community pharmacists, risk factors, decision trees, statistical learning

## Abstract

**Purpose:** The early detection of Mild Cognitive Impairment (MCI) is essential in aging societies where dementia is becoming a common manifestation among the elderly. Thus our aim is to develop a decision tree to discriminate individuals at risk of MCI among non-institutionalized elderly users of community pharmacy. A more clinically and patient-oriented role of the community pharmacist in primary care makes the dispensation of medication an adequate situation for an effective, rapid, easy, and reproducible screening of MCI.

**Methods:** A cross-sectional study was conducted with 728 non-institutionalized participants older than 65. A total of 167 variables were collected such as age, gender, educational attainment, daily sleep duration, reading frequency, subjective memory complaint, and medication. Two screening tests were used to detect possible MCI: Short Portable Mental State Questionnaire (SPMSQ) and the Mini-Mental State Examination (MMSE). Participants classified as positive were referred to clinical diagnosis. A decision tree and predictive models are presented as a result of applying techniques of machine learning for a more efficient enrollment.

**Results:** One hundred and twenty-eight participants (17.4%) scored positive on MCI tests. A recursive partitioning algorithm with the most significant variables determined that the most relevant for the decision tree are: female sex, sleeping more than 9 h daily, age higher than 79 years as risk factors, and reading frequency. Moreover, psychoanaleptics, nootropics, and antidepressants, and anti-inflammatory drugs achieve a high score of importance according to the predictive algorithms. Furthermore, results obtained from these algorithms agree with the current research on MCI.

**Conclusion:** Lifestyle-related factors such as sleep duration and the lack of reading habits are associated with the presence of positive in MCI test. Moreover, we have depicted how machine learning provides a sound methodology to produce tools for early detection of MCI in community pharmacy.

**Impact of findings on practice:** The community of pharmacists provided with adequate tools could develop a crucial task in the early detection of MCI to redirect them immediately to the specialists in neurology or psychiatry. Pharmacists are one of the most accessible and regularly visited health care professionals and they can play a vital role in early detection of MCI.

## 1. Introduction

The number of people with dementia is increasing due to a higher life expectancy, becoming one of the main issues in public health and demanding effective prevention measures. Indeed, according to the study provided by Prince et al. (2016), in 2015 there were 46.8 million people older than 60 who suffer from dementia worldwide and they will reach 131.5 million in 2050.

Especially, Alzheimer's Disease (AD) should be taken into consideration to being one of the most prevalent diseases related to dementia. For instance, its prevalence in south Europe is 6.88% with a significant difference between men (3.31%) and women (7.13%). The prevalence of AD increases with age, varying from 0.97% for 65–74 and 22.53% for older than 85 years old (Niu et al., 2017).

Mild Cognitive Impairment (MCI), a cognitive disturbance associated with age, it is a transitional stage between aging and dementia (Petersen et al., 1999). According to estimation performed by Petersen et al. (2018), the MCI prevalence is 8.4% for 65–69 and reaching 25.2% for older than 80 years old. As expected, the prevalence of MCI is greater than AD in all age ranges, being considered MCI a previous stage of the AD. More specifically, people with MCI have a higher risk of suffering from AD, presenting an annual conversion rate to dementia that varies between 10 and 12%, whereas the annual conversion rate is between 1 and 2% among the general population (Cornutiu, 2015). Therefore, MCI is rapidly becoming one of the most common clinical manifestations affecting the elderly and health practitioners should contribute to its early detection.

The cognitive ability that more frequently is affected by age and disease is the memory, which is the medium through our past experiences remain and it is retrieved in the present. In most of the cases, MCI patients are aware of their memory lapses at the start of a cognitive impairment but the subjective evaluation of the individual over the operation of memory itself cannot reflect an accurate assessment of the existence of a deficit of real memory. For an early assessment of a cognitive decline, corroboration by a caregiver/informant and measuring by psychometric tests scientifically validated are required.

Apart from genetic factors, demographic and lifestyle-related characteristics are also associated with this disease (Climent et al., 2013). Among these relevant lifestyle variables for cognitive impairment, some studies have identified daily and/or night sleep duration as a risk factor, which can motivate cognitive decline (Faubel et al., 2009; Benito-León et al., 2013; Ramos et al., 2013; Gabelle et al., 2017).

Furthermore, daily hours of sleep may be influenced by the consumption of drugs such as benzodiazepines (BZD) since they are the most prescribed drugs to treat insomnia. It is estimated that 20–25% of the elderly consume them (Fernández et al., 2008) and nearly three-quarters of the elderly who use them are chronic users (Velert Vila et al., 2012). The BZD may cause adverse effects related to neurological disorders that could be added to the basic cognitive impairment in the elderly (Ranstam et al., 1997; Airagnes et al., 2016).

Moreover, medications with strong anticholinergic side effects, such as first-generation antihistamines, tricyclic antidepressants, bronchodilators, antispasmodics, antiemetics, and urinary incontinence antimuscarinics are well-known drugs for causing acute cognitive impairment (Collamati et al., 2016). When it comes to related variables to MCI, depression is also acknowledged as a risk factor for dementia (Katon et al., 2012). Individuals with MCI are twice as likely to have been exposed to antidepressant drugs compared to those individuals without MCI (Moraros et al., 2017).

Unfortunately, none of the drugs tested to date in clinical trials in order to change the course of the disease have shown effective results in AD dementia. Therefore, in the absence of a cure for dementia, and given that current pharmacological treatments are ineffective when the disease has developed, many intervention studies are currently moving their focus to cognitively healthy individuals in risk of developing AD as only around 50% of people living with dementia receive a diagnosis(Prince et al., 2016).

Consequently, nowadays the challenge of prevention is focused on early detection (Crous-Bou et al., 2017). Furthermore, the subjective cognitive decline in memory precedes objectively cognitive symptoms of AD and promise as a non-invasive, inexpensive and preclinical indicator of MCI (Jessen et al., 2014; Rabin et al., 2017).

The role of the community pharmacist in primary care has been undergoing a change in Spain, it has become more clinically and patient-oriented. Task-shifting and task-sharing with primary care services will be a core strategy for improving diagnosis and continuing care. Increasing the role of primary care services may be up to 40% cheaper than specialist care in high-income countries. Task-shifting is defined as delegating selected tasks to existing or new health professional cadres with either less training or narrowly tailored training. This may involve creating new professional roles, so tasks can be shifted from workers with more general training to workers with specific training for a particular task (Prince et al., 2016).

Thus, this research study presents how the community pharmacist can develop a crucial task in screening potential MCI patients at early stages to redirect them immediately to the medical services (neurology or psychiatry) for a diagnosis. Specifically, the aim of the present study was to develop a prevention tool for an effective improvement in the detection of MCI by community pharmacies among non-institutionalized people.

Through the identification of risk factors associated with the presence of MCI in the elderly and the application of data-driven models, based on machine learning algorithms, a decision tree and a predictive tool have been developed in order to increase the efficiency of the screening methodology, discriminating which features play an important role for an immediate MCI detection. Therefore, we intend to increase the success rate in early identifying people with MCI, thus the design of the screening paradigm is focused toward obtaining a greater number of positives in the detection tests. In our opinion, one of the most appropriated location to carry out this screening is the community pharmacies, being our aspiration to develop a model framework of cognitive impairment management designed to improve care, enhance communication and collaboration among patients and providers, and optimize the screening for early detection of MCI.

## 2. Materials and methods

### 2.1. Study design

A cross-sectional study to detect cognitive impairment and potential associated risk factors in population aged 65 years and older was designed. Accordingly, the estimated sample size for a prevalence of cognitive impairment of 15±3% with a 95% confidence interval is 541 people; with an increase of 10% due to losses were 600 people.

### 2.2. Participants

**Figure 1:**
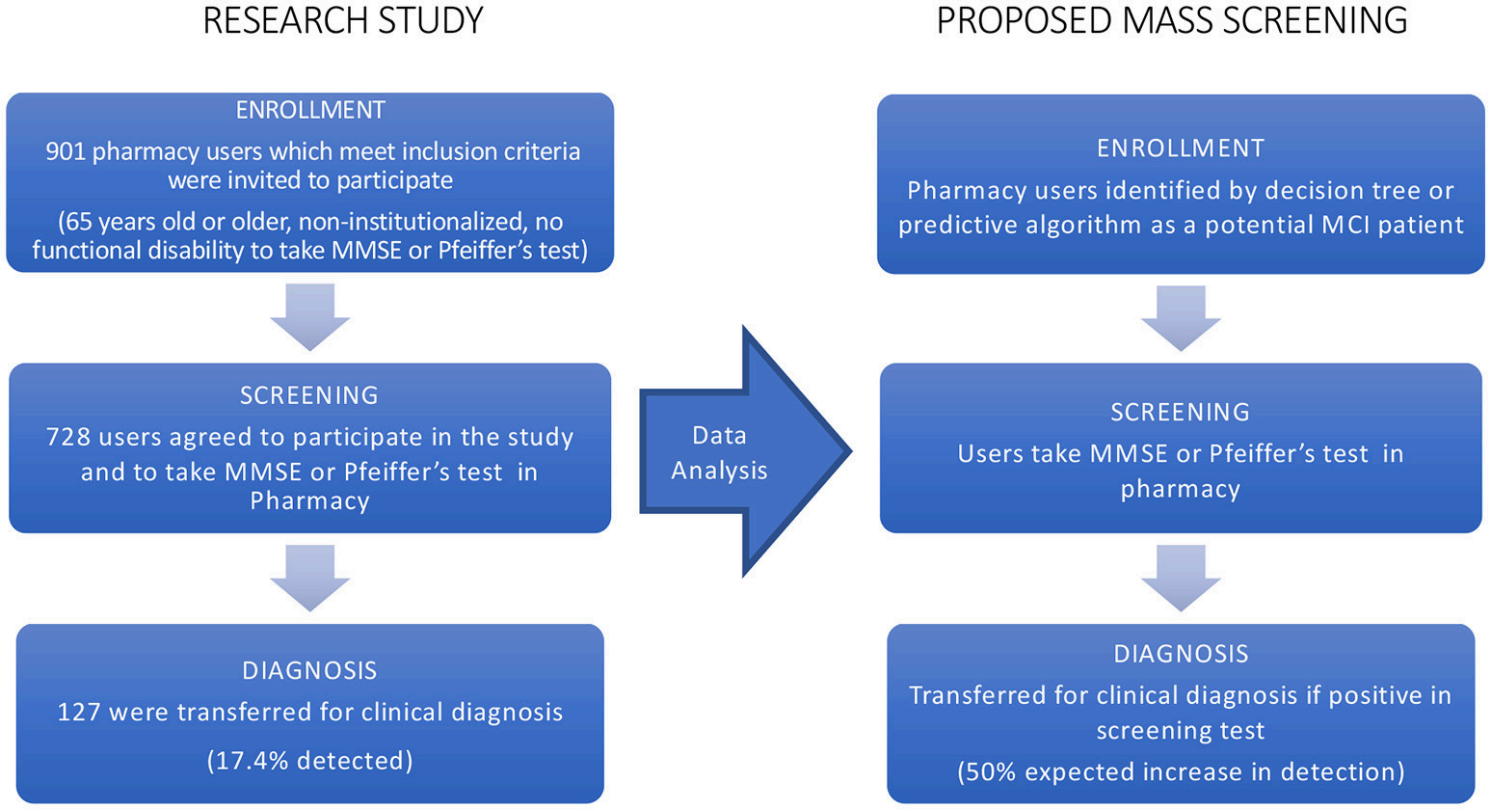
Flowchart of the research study. The flow on the left represents the research study whose data were applied machine learning algorithms. As a result of these techniques, a mass enrollment is proposed for early detection of MCI and the flowchart of the procedure is displayed on the right.

The present project has been carried out at the University CEU Cardenal Herrera together with community pharmacies in the region of Valencia (Spain) associated to SEFAC (*Spanish Society of Family and Community Pharmacy*). The study was conducted in 14 pharmacies from the Comunidad Valenciana (Spain), having each one different socio-economical characteristics: little town, countryside near a big city, different urban areas: central, semi-peripheral, and peripheral, referring to different socioeconomic levels. Subjects data were collected during 1 year, through structured personal interviews with the participants, which lasted approximately half an hour per individual. Additionally, every patient was subsequently controlled for 3 months. Inclusion criteria were non-institutionalized people aged 65 years or older who went regularly to the pharmacy and wished to participate in the present study. Conversely, exclusion criteria were people who had any difficulty to perform evaluation tests (blind, deaf,…) or people who were in treatment for dementia. Pharmacists invited users, which meet these criteria, to participate as it is depicted in Figure 1.

### 2.3. Study variables

A thorough search including terms as “cognitive impairment” and “risk factors” was conducted by using PubMed and the Cochrane systematic reviews before starting the study with the purpose of compiling the greatest number of factors that appear in the scientific literature as possible characteristics related to cognitive impairment.

Specifically in the Cochrane database, it was searched for all articles with the term “cognitive impairment” or “dementia” and in PubMed articles with the terms of: “sex,” “education level,” “cognitive activities,” “physical activity,” “diabetes,” “ hypertension,” “cholesterol,” “depression,” “obesity,” “smoking,” “alcohol,” “sleep,” “diet,” and “economic conditions” in combination with cognitive impairment or dementia too. Hence, a questionnaire was elaborated with the variables at the bibliographic review present some evidence of the relationship with cognitive deterioration or dementia.

Therefore, as a result of this search, the independent variables were age, gender, Body Mass Index (BMI—weight and height were measured directly), education level, daily sleep duration, consumption of drugs, especially benzodiazepines, etc. (among a total of 167 variables). The consumption of drugs was collected following the Dader's methodology (Salazar-Ospina et al., 2014).

The ATC (Anatomical Therapeutic Chemical) code was used to classify the drugs and the collaborating center assigned this code by WHO Collaborating Centre for Drugs Statistics Methodology (2018).

Moreover, the educational attainment was classified into four categories: Incomplete primary, primary schooling, secondary schooling, and graduated. We used the classification of occupations by social classes proposed by the Spanish Society of Epidemiology (Regidor, 2001) with the purpose of knowing their cognitive reserve although at the time of screening everyone was retired. The concept of cognitive reserve suggests that lifetime exposures including educational and occupational attainment, and leisure activities in late life, can increase this reserve. There is a reduced risk of developing AD in individuals with higher educational or occupational attainment (Stern, 2012).

Finally, for daily sleep duration, subjects reported the total number of daily hours they used to sleep, both during the night as during the day and if there had been changes during the last year in the sleep pattern, without changes in the medication, to be sure that is wasn't the effect of drug use.

All collected variablescan be seen in the Supplementary Materials.

### 2.4. Cognitive impairment assessment

Two tests were used to detect the presence of cognitive impairment, as the dependent variable, of participants under study. Specifically, such screening tests were the Spanish version of the Short Portable Mental State Questionnaire (SPMSQ) (Pfeiffer, 1975) and the Mini-Mental State Examination for Spanish speaking communities (MMSE, NORMACODEM) (Folstein et al., 1975; Blesa et al., 2001). With respect to the choice of these tests, it is known that the evaluation of cognitive impairment in the Spanish elderly is conditioned by the fact that most of the neuropsychological tests are influenced by the educational level. Given that the target population lived postwar during their childhood with serious socioeconomic problems and poor schooling, therefore there is a high percentage of illiteracy. Consequently, in this study, we used two screening tests that are not influenced by the level of schooling.

Study participants were considered cognitive impaired when at least one of the following criteria was met:

- Their scores in SPMSQ were 4 or more points in the case of illiterate participants and 3 or more points for other subjects.
- Less than or equal to 24 points in the corrected MMSE test.

The MMSE, Blesa's NORMACODEM version, has a maximum score of 30 and an additional maximum bonus of +2, in individuals with years of schooling ≤8 years and with an age > 75 years. The cut-off point is set at 24/25 once corrections are made for age and level of schooling (Blesa et al., 2001). In such a way that cases of MCI are considered to those participants with a score less than or equal to 24. The sensitivity of MNSE is 87 % and its specificity is 89 % for AD. The presence of cognitive impairment in illiterate participants was only tested by the SPMSQ test. These tests are selected because are routinely used in memory clinics. The MMSE is a measure of general cognitive function and includes orientation to time and place, attention and calculation, language, and memory.

A specialized physician, this time a neurologist, trained the pharmacists on the use of memory screening instruments. About the participants in the screening, they were invited to answer a standardized interview including questions about demographics, health, and medication. Additionally, services were offered to patients identified within the community pharmacy by appointments and the positive cases in the MCI screening were referred to the medical specialist (a neurologist) for their clinical diagnosis, which is the last step of the chart flow of the research study as it can be seen in Figure 1.

### 2.5. Statistical analysis and preprocessing

An initial descriptive analysis was conducted. Consequently, for categorical variables, a contingency table, the *p*-value of a univariate logistic regression with response variable the screening for MCI, sample odds ratio ($\hat{\text{OR}}$) and a 95% confidence interval of odds ratio are shown in Table 1. Moreover, it has been taking into account variables where there is at least one statistically significant category (α < 0.01).

**Table 1:** Description of odds ratios for qualitative variables with at least one category statistically significant with a univariate logistic regression for a significance level α = 0.01.

**Variable**	**No N(%)**	**Yes N(%)**	***p-value***	**OR ( 95% CI)**
**READING**				
Never	163 (27.12)	70 (55.12)		
Sometimes	174 (28.95)	31 (24.41)	2.7e-04	0.41 (0.26,0.67)
Daily	264 (43.93)	26 (20.47)	4.0e-09	0.23 (0.14,0.37)
**MEMORY COMPLAINT**				
No	372 (61.9)	43 (33.86)		
Yes	229 (38.1)	84 (66.14)	1.9e-08	3.17 (2.12,4.75)
**N06**				
No	514 (85.52)	83 (65.35)		
Yes	87 (14.48)	44 (34.65)	2e-07	3.13 (2.04,4.82)
**EDUCATIONAL ATTAINMENT**				
Incomplete Primary	39 (6.49)	30 (23.62)		
Primary	378 (62.9)	74 (58.27)	6.0e-07	0.25 (0.15,0.44)
Secondary	134 (22.3)	20 (15.75)	1.5e-06	0.19 (0.1,0.38)
Tertiary	50 (8.32)	3 (2.36)	7.1e-05	0.08 (0.02,0.27)
**SEX**				
Male	260 (43.26)	32 (25.2)		
Female	341 (56.74)	95 (74.8)	2.1e-4	2.26 (1.47,3.49)
**DEPRESSION**				
No	506 (84.19)	89 (70.08)		
Yes	95 (15.81)	38 (29.92)	2.4e-4	2.27 (1.47,3.53)
**M01**				
No	467 (77.7)	114 (89.76)		
Yes	134 (22.3)	13 (10.24)	2.8e-3	0.4 (0.22,0.73)
**JOB**				
6	261 (43.43)	77 (60.63)		
5	94 (15.64)	20 (15.75)	0.2402	0.72 (0.42,1.24)
4	114 (18.97)	18 (14.17)	0.0282	0.54 (0.31,0.94)
3	79 (13.14)	7 (5.51)	0.0038	0.3 (0.13,0.68)
2	31 (5.16)	5 (3.94)	0.2263	0.55 (0.21,1.45)
1	22 (3.66)	0 (0)	0.9761	0 (0, Inf)

Nevertheless, for ordinal variables, dummy variables contrast has been employed for logistic regression whereas contrasts with orthogonal polynomials, which convert these qualitative to quantitative ones Chambers and Hastie (1991, p. 32–37), were applied according to the machine learning algorithms.

Subsequently, for quantitative variables, mean and standard deviation were calculated in the group, being presented in Table 2 the variables which are statistically significant. These variables are categorized to analyze odds ratios (OR) and their 95% confidence intervals (CI) in order to estimate the association between cognitive impairment and the different categories.

**Table 2:** Description of significant quantitative variables for a univariate logistic regression with a significance level α = 0.01.

**Variable**	**na**	**No $\bar{x}\left(s\right)$**	**Yes $\bar{x}\left(s\right)$**	***p-value***	**OR ( 95% CI)**
Height $\left(\bar{x},s\right)$	1	1.62 (0.08)	1.59 (0.0 9)	3.2e-05	
P20-P80 N(%)		434 (72.21)	77 (60.63)		
P20 N(%)		89 (14.81)	41 (32.28)	2.4e-05	2.6 (1.67,4.04)
P80 N(%)		78 (12.98)	8 (6.3)	1.6e-01	0.58 (0.27,1.24)
Overnight sleeping time $\left(\bar{x},s\right)$	0	7.07 (1.57)	7.72 (1.83)	6.1e-05	
6-9 N(%)		466 (77.54)	90 (70.87)		
< 6 N(%)		99 (16.47)	16 (12.6)	0.54318	0.84 (0.47,1.49)
>9 N(%)		36 (5.99)	21 (16.54)	0.00021	3.02 (1.69,5.41)
Sleeping time $\left(\bar{x},s\right)$	0	7.62 (1.75)	8.21 (1.93)	0.001	
6-8h N(%)		411 (68.39)	66 (51.97)		
< 6h N(%)		51 (8.49)	10 (7.87)	5.9e-01	1.22 (0.59,2.52)
>9h N(%)		139 (23.13)	51 (40.16)	8.8e-05	2.28 (1.51,3.45)
Age $\left(\bar{x},s\right)$	0	74.17 (6.28)	75.84 (6.77)	0.0079	
65-69 N(%)		162 (26.96)	29 (22.83)		
70-74 N(%)		182 (30.28)	26 (20.47)	0.4380	0.8 (0.45,1.41)
75-79 N(%)		130 (21.63)	37 (29.13)	0.0912	1.59 (0.93,2.72)
80-84 N(%)		88 (14.64)	16 (12.6)	0.9633	1.02 (0.52,1.97)
85+ N(%)		39 (6.49)	19 (14.96)	0.0037	2.72 (1.38,5.35)

*These variables have been categorized into several groups and the sample odds ratio and 95%confidence interval for these groups*.

### 2.6. Development of decision trees

With the aim of developing a fast and functional tool to decide which subject has to undergo the SPMSQ or the MMSE test, i.e., a quick cognitive impairment screening with the maximum accuracy, decision tree algorithms were constructed. In fact, a binary classification tree model is proposed. Here, the binary term refers to splitting the data into two major outcomes of interest: possible cognitive impairment that means the subject has to carry out the test or the contrary, i.e., low probability of obtaining a scoring compatible with cognitive impairment, thus no necessity of performing such tests.

Trees are simple and powerful machine learning models, that can generate a set of conditions highly interpretable and straightforward to implement. Additionally, as a consequence of the logic of their construction, they can handle many types of predictors without the need for preprocessing. Furthermore, such models can effectively handle missing data and implicitly conduct a feature selection, desirable characteristics for numerous real-life applications (Hastie et al., 2001; Kuhn and Johnson, 2013).

To develop such models training and testing phases must be carried out, in the present project by means of using caret package (Kuhn, 2008). Thus, for the purpose of model acceptance, we randomly selected an 80% of the data for training the models and the remaining 20% as a test set for validation. The discriminating ability of the models was assessed by using the Receiver Operating Characteristic (ROC) curve and the Area Under the ROC curve (AUC) to compare different models. Additionally, the random seed has been fixed to allow reproducibility of the study too. These characteristics establish the general framework to develop a decision tree and a predictive tree for the next sections.

#### 2.6.1. Discriminant decision tree

With the purpose of developing an easy-to-interpret decision tree, the recursive partitioning (known as “rpart,” a version of CART library available at R foundation, http://cran.rproject.org/web/packages/rpart/rpart.pdf) was employed to create a binary decision tree, splitting the data into two major outcomes of interest in relationship with the presence of cognitive impairment or not.

The method uses a splitting rule built around the notion of purity. A node in the tree is defined as pure when all the elements belong to one class. However, when there is impurity in the node, a split occurs to maximize the reduction in impurity. In some cases, the split may be biased toward attributes that contain many different ordinal levels or scales. Consequently, the selection of an attribute as the root node may vary according to the splitting rule and the scaling of the attribute (Phan et al., 2017).

Furthermore, to construct the decision tree the prior probabilities have been established to 0.83 for the negative class and 0.17 for the positive (i.e., cognitive impairment) one. Accordingly with the proportion of the disease found in our dataset. Moreover, the loss matrix has been also parametrized in order to benefit the algorithm classification for a superior sensitivity, as the most important is to detect the maximum true positives, i.e., people with cognitive impairment that the algorithm predicts they have to undergo the questionnaire and it will be positive, but also to reduce the false negatives. The splitting rule has been settled to “gini,” with the objective of using the Gini index in order to define the purity as a measure of accuracy when data is partitioned by the algorithm (Kuhn and Johnson, 2013).

Additionally, as the dataset is imbalanced, such dataset problem has been overcome by using a technique of downsampling. Such technique is a straightforward method to reduce the impact of class imbalance in the training set, reducing randomly the number of samples to balance across classes, and this improved substantially the accuracy at the training phase.

Moreover, with the intention to minimize an overfitting problem, a resampling technique has been employed to estimate the efficacy of the model, thus K-fold cross-validation method has been selected (Kuhn and Johnson, 2013). It involves splitting the dataset into k-subsets, 10-fold for the present study. Thus, samples are randomly partitioned into 10 subsets. For each subset is held out while the model is trained on all other subsets. This process is completed until accuracy is determined for each instance in the dataset, and an overall accuracy estimate is provided.

#### 2.6.2. Predictive tree model

Additionally, three of the most widespread and powerful techniques of classification trees have been applied to the dataset with the goal of generating models which provide probabilities of a patient being affected by mild cognitive impairment. These models achieve a higher predictive accuracy, but the reasons why individuals have been assigned their probabilities are concealed by very complex models. Accordingly, these models bring us the possibility of understanding which are the variables most relevant to predict a positive/negative result in the mild cognitive impairment tests. Notwithstanding, they don't bring the possibility of obtaining an explanatory decision tree as above. Hence, the complementarity of such models for the present work.

Specifically, the algorithms employed to generate these models are: Random Forest (RF) (Breiman, 2001), Extreme Gradient Boosting (XGBoost) (Chen and Guestrin, 2016), and Generalized Boosted Regression Tree (GBM) (Friedman, 2001, 2002).

Moreover, in order to profit the goodness of all them, an ensemble of all the above has also been constructed, which produced an enhanced final prediction accuracy. It was deployed by combining model predictions into ensemble predictions. Therefore, the caretEnsemble package (available at from R foundation, https://cran.r-project.org/web/packages/caretEnsemble/caretEnsemble.pdf) has been employed for this task. The ensemble technique provides a method to integrate the characteristics each model is able to capture in a unique model.

Furthermore, all models have been trained under the same resampling parameters, using this time a repeated cross-validation ten times and a GLM (Generalized Linear Model) to create a final linear blend of models.

### 2.7. Ethical approval

This study was reviewed and approved by the Research Ethics Committee of Universidad CEU Cardenal Herrera (approval no. CEI11/001) on March 2011. All subjects gave written informed consent in accordance with the Declaration of Helsinki.

## 3. Results

### 3.1. Demographic characteristics

A total of 901 people between 65 and 89 years old were asked if they wanted to participate in the study. One hundred and seventy-two declined to participate (98 women and 74 men). Therefore, 728 subjects participated in the study, 436 women (59.9%) and 292 men (40.1%). The average age among participants was 74.5 ± 6.4 years old (± standard deviation, SD). In general, the educational attainment was biased to elementary school: Incomplete primary (9.5%), primary schooling (62.1%), secondary schooling (21.2%), and graduate (7.3%).

Regarding the BMI, the mean value among participants was 27.3 ± 4.3 Kg/m^2^. Two hundred and thirty-three subjects (32.0%) had normal weight (BMI < 25 Kg/m^2^) whereas 322 (44.3%) and 172 (23.7%) had overweight (25≤BMI < 30 Kg/m^2^) and obesity (BMI≥30 Kg/m^2^), respectively.

The most frequently consumed drugs included: antihypertensive drugs (43.5%) preferably angiotensin II antagonists (31.7%); lipid modifying agents (38.7%) as statines (34%); drugs for peptic ulcer (35.4%), where proton pump inhibitors (33.1%) were the most employed; anxiolytics (25.8%); antithrombotic agents (22.9%); antiinflammatory non-steroids drugs (20.2%) and analgesic (19.3%); drugs used in diabetes (19.9%) in a majority way (18.3%) oral antidiabetic drugs as biguanides (8.79%). Eighteen percent take psychoanaleptics, drugs as antidepressants (13.5%) like selective serotonin reuptake inhibitors (9.4%). Diuretics are used by 17.8% as well as high ceiling diuretics (9.3%). Moreover, other drugs consumed were vasoprotective (16.3%), beta-blockers (13.7%), drugs utilized in cardiac therapy (12.0%), calcium channel blockers (11.8%), ophthalmological and otological preparations (11.6%), bisphosphonates (10.8%), and drugs used in benign prostatic hypertrophy (10%).

The mean daily sleep duration was 7.7 ± 1.8 h. Nevertheless, 190 of the study participants (26.1%) usually sleep 9 h or more per day.

### 3.2. Logistic regression analysis

One hundred and twenty-seven participants (17.4%) had scores in the screening tests compatible with the presence of cognitive impairment. The distribution of positive cases depending on the age does not show a significant difference with the first interval, but for individuals older than 85. The percentages of positive on the screening depending on the age interval are: 65–69 (15.18%), 70–74 (12.5%), 75–79 (22.16%), 80–84 (16.00%), and older than 85 (32.76%). The mean score in the SPMSQ test among impaired participants was 1.19±1.36 while the mean score in the MMSE test was 26.91±3.19.

Previously described in the section 2, the univariate logistic analysis in Tables 1, 2 makes evident that there exist association with socio-demographic characteristics, drugs prescription, and a subjective self-assessment with a positive outcome in MCI screening .

In this univariate logistic analysis, the variables presenting the highest association with being positive in MCI screening are the frequency of reading and subjective memory complaint. Firstly, there is a very highly significant association between not being engaged in reading activities and the chances of a positive MCI screening. In the second place, the role of memory complaint, as a sign of being a positive testing in MCI screening, is beyond a doubt, being the *p*-value less than 10^−7^. This suggests that the lack of reading and memory complaint be primary indicators for a discriminant tool to locate population groups at risk of MCI.

According to Table 2 for quantitative variables, the height is the most significant variable of this type. Nonetheless, this variable is very correlated to the sex of the participant which is also among the significant variables in the analysis. However, this association vanishes If the analysis is repeated splitting into male and female participants, suggesting that the sex be the variable to be taken into account to design any discriminative tool.

On the other hand, obesity was found as a risk factor for cognitive impairment with statistical significance when compared with normal weight (OR 1.78; 95% CI 1.06–2.99 and *p*-value = 0.029).

When comparing with the group sleeping from 6 to 9 daily hours, it was observed that sleep for more than 9 h per day was a risk factor for cognitive impairment with a high statistical significance (OR 2.28; 95% CI 1.51–3.45 and *p*-value < 10^−4^). Furthermore, the same association is found for the nocturnal sleep duration.

Regarding educational attainment, all the categories show a highly significant association with a positive in MCI screening with respect to the no completion of the primary school with all the *p*-values less than 10^−4^. The variable job classification has shown association with being positive in MCI screening, but it is very correlated to the educational level which is more significant according to this data.

Few ATC codes have shown a significant difference with respect to the positive cases on the screening. The most relevant groups according to the *p*-value are N06 and M01. Therefore, the first group includes drugs for the treatment of depression and memory enhancing drugs presenting a positive association with the screening of MCI. Notwithstanding, it is worth mentioning that there is a quantitative variable indicating if the patient suffered from depression which is significant (*p*-value < 10^−3^) . In contrast, the second group M01 are anti-inflammatory drugs displayed a negative association with the screening of MCI.

### 3.3. Decision tree models analysis

In order to develop the decision tree, some of the most significant variables, obtained through the univariate logistic regression results, have been chosen. The criterion for selection was that they have to be predictive of cognitive impairment, through checking the odds ratios and *p*-values, and should also be straightforward to be recollected at pharmacies, i.e., easy decision by the pharmacist to recommend the person to carry out the test concerning to the SPMSQ or the MMSE test having a high probability of positive result.

**Figure 2:**
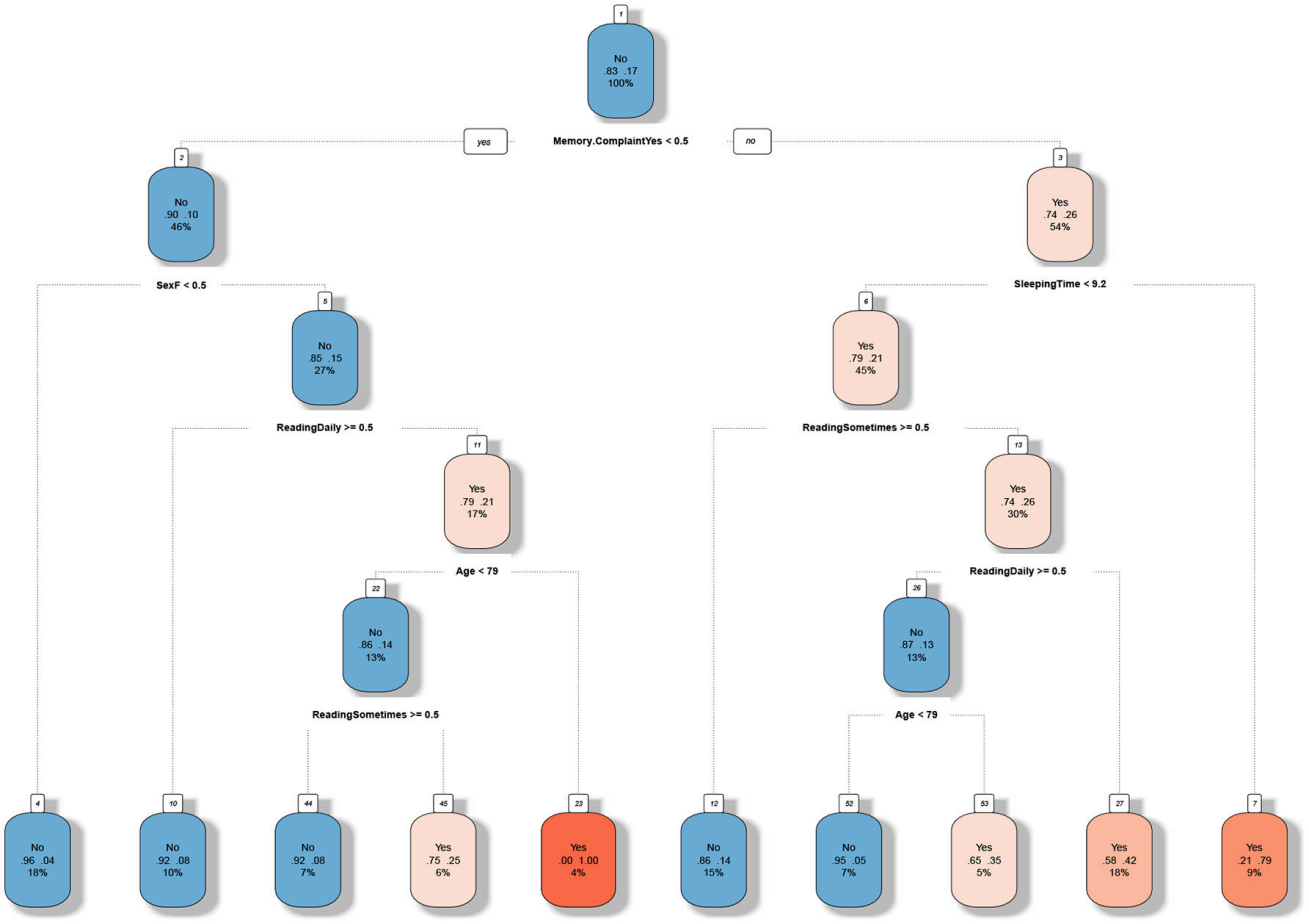
Tree developed with a recursive partitioning algorithm using a training set. For every box, the first line is whether or not the user is classified to be in risk of MCI. The second line consists in two numbers, which indicates the estimated probability of being positive in MCI. The last line in the box is the percentage of the data fulfilling these conditions. The text below the box is the question corresponding to the next split. The warmer is the color of the box, the more likely is a user in that node to be positive in MCI.

Accordingly, variables selected to train the model have been sex, age, educational attainment, reading habits, sleeping total time, depression, and memory complaint. Notwithstanding, after developing the decision tree, the algorithm has considered as the highest decisive the memory complaint followed by sex, sleeping total time, reading habits, and age, as it can be seen at Figure 2.

The tree has a final AUC of 0.763, a Sensitivity of 0.76 and Specificity of 0.70, due to the tree has been constructed to minimize the false negatives, as the primary objective. Consequently, it means some accuracy has been sacrificed in order to identify the maximum of people having a positive result of cognitive impairment performing the tests. That also means the algorithm recommends to some subjects to perform the tests although a priori the probability of a positive result is low. Therefore, the accuracy of the tree obtained from the confusion matrix calculation is 0.71 with a 95% CI:(0.6293, 0.7826).

Final nodes define the rules the algorithm has been extracted from data. Information at nodes identify with “yes” or “no” if the subject should undergo the tests; which are the probabilities of obtaining a negative and positive result; and finally the percentage of individuals that represent such rule in the dataset.

As it can be seen in Figure 2, the tree shows which are the highest critical paths to the MCI presence. Hot colors are more present on the right path, what means if memory complaint is present the probability of positive result in tests is high. But also, the fact of having a habit of reading seems to prevent the development of the disease, what is a lifestyle conduct that in some cases can be changed. Contrarily, cold or blue paths let us know which characteristics have a low probability of positive result at tests, as for example, not having memory complaints in relationship with sex and age. In literature, we can find such features as significant factors studied separately. Nevertheless, the tree we present tries to integrate some of them with the purpose of having an interpretable decision tree for fast screening as mentioned before.

### 3.4. Cognitive impairment prediction

With the aim to realize an improved precision in the prediction, additional powerful algorithms have been tested as presented above. The idea is to check if we can develop a predictive system with all the data we have collected from each participant. And consequently, understand better which are the most relevant factors to forecast the mild cognitive impairment. Trees give us the advantage of capturing non-linearities in data, thus all variables have been selected to train different models. The results showed that a higher prediction accuracy is achievable by each of the advanced models trained, but is also possible to be augmented by means of an ensemble of all of one.

**Figure 3:**
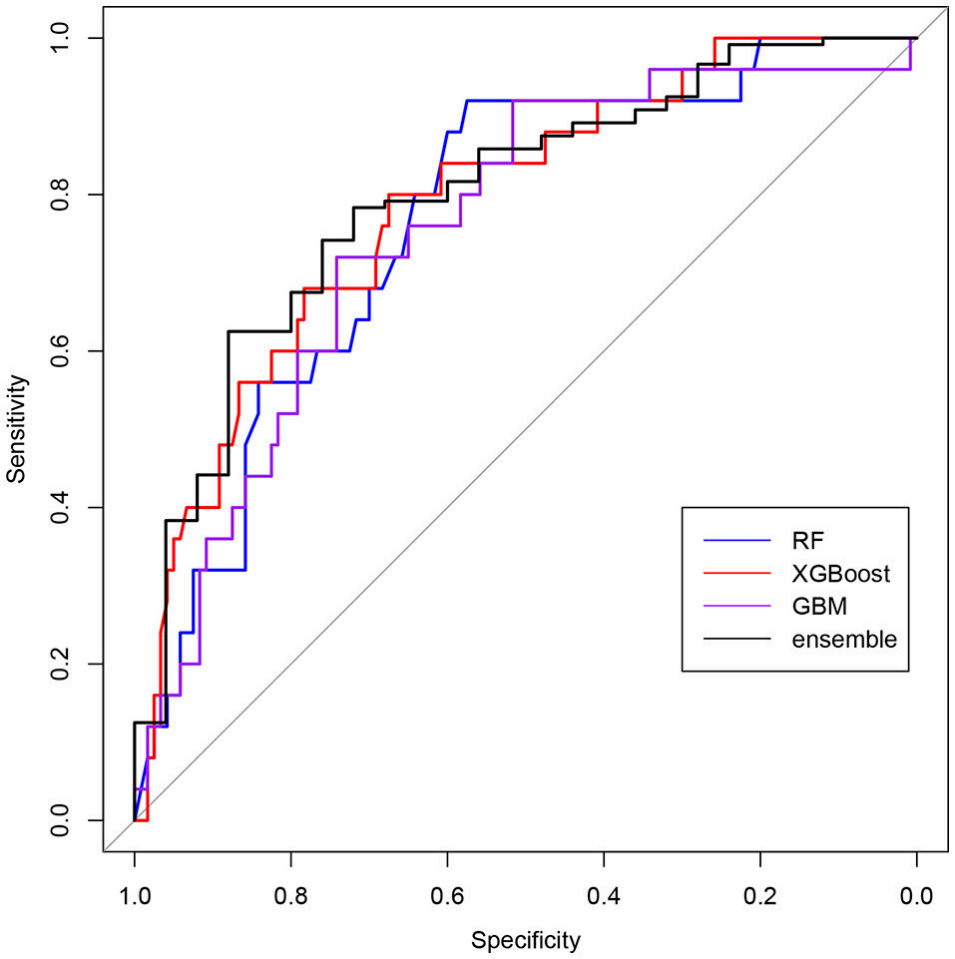
ROC curves of the predictive models for the test set. Models assign a probability to every user and the sensitivity and specificity are calculated for all possible cut–off points. Blue corresponds to Random Forest (RF), red is Extreme Gradient Boosting (XGBoost), purple to Stochastic Gradient Boosting (GBM), and black to the ensemble of models.

This can be seen at Figure 3 that shows the ROC results for the different models. Moreover, the final AUC in training and testing and their confidence intervals of the different models is also described at Table 3. Table 4 shows models correlations which were low, showing their predictions are fairly uncorrelated nevertheless their overall accuracy is similar, making such models very good candidates for an ensemble one. Hence, the final model has an AUC of 0.8007 in predicting the mild cognitive impairment evaluated through the MCI tests.

**Table 3:** Comparison of the area under ROC curve (AUC) for several models.

**Method**	**Estimated AUC in training**	**AUC in test**	**95% CI in test**
Random forest	0.6987	0.7667	(0.6709, 0.8624)
Extreme gradient boosting	0.6655	0.7873	(0.691, 0.8837)
GBM	0.6634	0.7557	(0.6532, 0.8582)
Ensemble model	0.7471	0.8007	(0.7044, 0.8969)

*The parameters deployed by each model are: Random Forest model (with parameter mtry = 23), Extreme Gradient Boosting (with the parameters: nrounds = 30, max depth = 1, eta = 0.45, gamma = 0, colsample bytree = 0.7 and min child weight = 1), and GBM (Stochastic Gradient Boosting ) (with parameters n.trees = 50, interaction.depth = 1, shrinkage = 0.1, and n.minobsinnode = 10). The final one the Ensemble Model: Random Forest (mtry = 169), Extreme Gradient Boosting (nrounds = 50, max depth = 1, eta = 0.3, gamma = 0, colsample bytree = 0.8, and min child weight = 1) and GBM ( n.trees = 50, interaction.depth = 3, shrinkage = 0.1, and n.minobsinnode = 10)*.

**Table 4:** Correlation of the models in the Ensemble Model: Random Forest (RF), Extreme Gradient Boosting (XGBoost), and Gradient Boosting Machine (GBM).

**Model/Correlation**	**GBM**	**RF**	**XGBoost**
GBM	1.0000000	0.31819462	0.45761406
RF	0.3181946	1.00000000	0.05869544
XGBoost	0.4576141	0.05869544	1.00000000

Additionally, the importance of the variables for the MCI prediction by each model is depicted at Table 5. It is shown which are the most decisive predictors of a set of 167 variables. Table 5 describes the 20 most crucial for the ensemble and their relevance to the other models. As it can be seen, demographic and lifestyle variables seem very discriminant, notwithstanding the consumption of drugs classified as N06, M01, and N06B (nootropics) appears also as influential.

**Table 5:** Importance of the variable scaled according the method varImp in the caret library for the complete data set.

**Important variables**	**Overall**	**GBM**	**RF**	**XGBoost**
Memory complaint	14.31	9.69	3.93	19.95
Overnight sleeping time	11.93	9.04	6.82	14.87
Educational attainment (linear contrast)	11.29	8.85	5.51	14.41
Reading (linear contrast)	10.73	9.75	5.35	13.33
Age	6.86	9.00	6.76	6.41
Sex	4.41	4.84	1.27	5.69
N06	4.12	3.14	1.38	5.55
M01	3.87	1.27	0.63	5.90
N06B	2.71	3.01	0.82	3.47
Job	2.40	1.74	2.00	2.72
Sleeping time	2.24	5.90	5.38	0.00
Games (linear contrast)	1.52	1.76	1.46	1.49
M01A	1.40	1.74	0.49	1.73
Smoking (linear contrast)	1.39	0.33	0.44	2.06
M02	1.02	0.31	0.39	1.47
C08	0.83	1.79	1.28	0.40
TV consumption	0.79	1.30	2.30	0.00
Day time nap	0.78	1.68	2.07	0.00
Educational attainment (cubic contrast)	0.64	2.94	0.85	0.00
Exercises (cubic contrast)	0.57	0.84	1.63	0.04

*Variables have been ordered depending on the importance in the ensemble model*.

**Figure 4:**
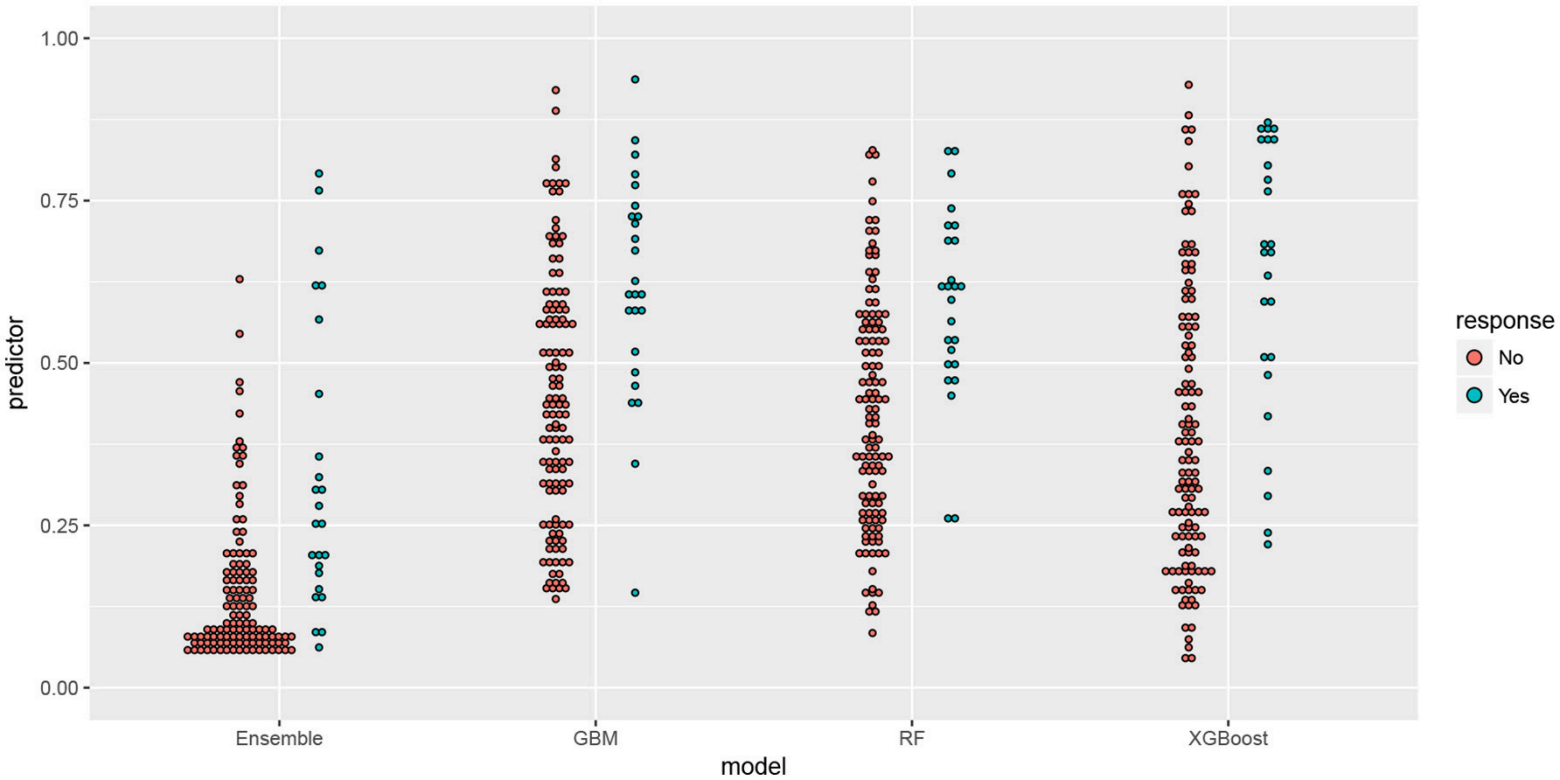
Comparison of predictor and response in the test set for predictive models seen in the article. Along the x-axis are the name of the model split into two groups: positive in MCI and negative in MCI according to the score in the test, on the y-axis is the probability (predictor) assigned by the model.

Furthermore, a comparison of the probabilities assigned by the predictive models in the test set is shown in Figure 4, being the individuals split into two groups depending on the result in the screening. The Random Forest and GBM algorithms have been able to assign a probability higher than average for most of the participants with positive screening, whereas XGBOOST has been able to distinguish a group of participants whose characteristics make them in risk of MCI.

## 4. Discussion

Mild Cognitive Impairment (MCI), the symptomatic predementia phase of the AD, is common in older populations and its prevalence increase with age, female sex and lower educational level. A review from Ward et al. (2012) establishes that the prevalence of MCI varies extensively across international studies, from around 3 to 42%. But small changes to elements of this criterion, as for example the threshold for impairment and the number of sub-threshold cognitive test results required, can significantly affect the prevalence of MCI reported (Sachdev et al., 2015).

Moreover, MCI symptom is also associated with reversible risk factors, including side effects of drugs, sleep, or depression among other. In this sense, an effective intervention to delay cognitive decline may be best targeted at the earliest symptomatic stage, such as the subjective memory complaint that precedes the MCI. This stage would presage the emergence symptoms 15 years prior to the diagnosis (Rabin et al., 2017). In fact, the guideline development, dissemination and implementation subcommittee of the American Academy of Neurology recommends an early and appropriate diagnosis and treating risk factors of MCI as the first step, because symptomatic treatment options are limited (Petersen et al., 2018). That is the goal of the present study, to maximize the detection rate of possible MCI cases in order to achieve an early diagnosis. Using 2 screening tests we have identified 17% of people at risk of MCI.

In addition to the cognitive complaint, longer sleep duration also predicts cognitive decline and is associated with MCI independently of major confounding factors. Sleep spindles have been intimately linked to overnight memory consolidation in healthy people. Hence, the importance of early detection of abnormal sleep-wake patterns in patients as a clinical indicator of neurodegenerative diseases to further propose sleep health strategies to prevent MCI (Gabelle et al., 2017; Naismith and Mowszowski, 2017).

On the other hand, some studies have demonstrated a substantial association between a low level of education and higher prevalence of AD. The theory of cognitive reserve proposes that brain pathology and age-related changes can be protected as a result of the way in which tasks are processed. Moreover, the discrepancy in clinical status between MCI and AD patients with a similar magnitude of brain pathology and comorbid medical disorders might be explained through the premorbid intellectual function (Osone et al., 2015). The above facts justify that the subjective memory complaint, the excessive hours of sleep and the habit of reading appear as factors to be taken into account in the screening process of MCI (Su et al., 2017)

Additionally, with respect to the drugs' consumption, the N06 group corresponding to antidepressants drugs and psychostimulants and nootropics agents (N06B) appears as influential. Such drugs emerge relevant in the predictive study in patients who use them, either because depression is the prelude to dementia or because of the anticholinergic effects of many of these drugs that can aggravate the cognitive decline. An over reporting of complaints in older adults with depression could be a consequence of a hypersensitivity to perceived cognitive failures. However, research suggests that the relationships between depression, MCI, and risk of cognitive decline are complex and these patients did not be excluded from cognitive screening. It has been suggested that late-life depression is associated with increased risk of all-cause dementia (da Silva et al., 2013; Diniz et al., 2013). Thus, special attention has to be taken to patients not diagnosed with dementia who use the N06B (nootropics) drugs group, as they usually suffer from subjective memory complaint.

Finally, another pharmacological group that has influence in the predictive model is the M01 group, that refers to non-steroidal anti-inflammatory drugs. Inflammation has been shown to play a key role in the early progression of the AD and several clinically-oriented epidemiological studies have indicated a lower incidence and progression of the AD in arthritis patients, who are treated with Nonsteroidal Anti-inflammatory (NSAID) drugs (Akiyama et al., 2000).

Hence, it is critical to find sensitive low-cost methods for early detection of individual therapeutic targeting of modifiable risk factors for dementia disease. In that direction data-driven models, based on the latest machine learning algorithms, give us an opportunity to develop decision trees in order to screen rapidly a target group with less cost and higher accuracy. As a result, these tools provide a cost-effective enrollment procedure (Figure 1) which could allow a mass screening in the target population. On the other hand, with the purpose of prediction, such models represent mathematically the problem, giving a novel and complementary approach to understanding better the disease, thus discoveries from data improve our knowledge.

Tree-based and rule-based models are conceptually simple yet powerful. They are common modeling tools for several reasons, although as far as we know never used for the purpose of the present research. Trees are simple to understand, interpret, and visualize and they can handle both numerical and categorical features natively. Furthermore, they are also robust to outliers and requires little data preparation. And additionally, they can model non-linearities in the data and can be trained quickly on large datasets.

We want to finish claiming that community pharmacists are one of the most accessible and regularly visited health care professionals in primary care, thus they can play a vital role in the early detection of cognitive impairment. Furthermore, MCI is more common in older age patients, which suffer from diseases such as hypertension, diabetes or depression. Such patients are intensive users of health services, and visit a pharmacist every 2 weeks. For that reason, cognitive memory screening can be easily incorporated into clinical service offerings in community pharmacy practice and provides a valuable opportunity to identify patients at risk to refer them to a psychiatrist or neurologist for diagnosis. Therefore, pharmacists are ideally placed to detect early signs of MCI (Stern, 2012).

## 5. Conclusion

A longer life expectancy is increasing the number of people with dementia, becoming one of the main issues in public health and demanding effective prevention measures. In that sense, the role of the community pharmacist in primary care has been undergoing a change in Spain, becoming more clinically and patient-oriented. Hence, it makes feasible the community pharmacist to develop a crucial task in detecting, at early stages, people with MCI to redirect them immediately to the medical services. Community pharmacists are one of the most accessible and regularly visited health care professionals in primary care.

Nevertheless, in order to perform a cost-effective screening of MCI new methodologies and tools become mandatory. In that direction decision tree models allow us to understand better a problem described from data, i.e., data-driven models. Thus the use of easily interpretable trees can enhance substantially the screening outcomes. In fact, preliminary results testing the developed tree in this paper showed an increase of around 50% of gain in the detection of people with MCI. Moreover, the use of advanced predictive trees helped us to complement a traditional technique, as the logistic regression, to describe the most influential factors predicting the MCI tests results, considering also the non-linearities in data. Such approaches give us the opportunity to continue studying MCI to detect it at pharmacies in a new preventive and proactive paradigm.

## Data availability statement

Datasets are available on request: The raw data supporting the conclusions of this manuscript will be made available by the authors, without undue reservation, to any qualified researcher.

## Author contributions

MC contributed to the acquisition of the data and write-up. JP and FM-A assisted in data analysis, interpretation, models development, and write-up. MG assisted in data collection and write-up. LM assisted in concept, design, critical revision, and write-up. All authors listed have made a substantial, and direct contribution to the work, and approved the final manuscript as submitted.

### Conflict of interest statement

The authors declare that the research was conducted in the absence of any commercial or financial relationships that could be construed as a potential conflict of interest.
