# Supplementary material for: Decision Tree for Early Detection of Cognitive Impairment by Community Pharmacists
Source: Front Pharmacol. 2018 Oct 29;9:1232. doi: 10.3389/fphar.2018.01232 (PMC6215965; doi:10.3389/fphar.2018.01232)
Supplement: Supplementary file 1 [file Table_1.PDF]

Supplementary Material:
Decision tree for early detection of cognitive impairment by community pharmacists

1 UNIVARIATE LOGISTIC REGRESSION FOR ALL VARIABLES

The column **variable** is the name of the variable in the data set and the indented values are the categories for quantitative variables, the column **NA** is the number of missing values, **No MCI** and **MCI** is the statistics for users without and with mild cognitive impairment (MCI), the following column is the **p-value** of the logistic regression and the last column$\hat{\beta}$ is the estimation of the coefficient of the logistic regression and its 95% CI. For quantitative variables are given sample mean ($\overline{x}$) and standard deviation (s) and for qualitative variables the frequency (N) and the percentage(%).

Table S1: Table with univariate logistic regression analysis for all the variables in the data set.

**variable**	**NA**	**No MCI**	**MCI**	**p-value**	$\hat{\beta}$ **95%CI**($\hat{\beta}$)
Sex	0				
M N(%)		260 (43.26)	32 (25.2)		
F N(%)		341 (56.74)	95 (74.8)	0.00021	0.82 (0.39,1.25)
Age ($\overline{x}, s$)	0	74.17 (6.28)	75.84 (6.77)	0.0079	0.04 (0.01,0.07)
Educational.Attainment	0				
Incomplete Primary N(%)		39 (6.49)	30 (23.62)		
Primary N(%)		378 (62.9)	74 (58.27)	6.0e-07	-1.37 (-1.91,-0.83)
Secondary N(%)		134 (22.3)	20 (15.75)	1.5e-06	-1.64 (-2.31,-0.97)
Tertiary N(%)		50 (8.32)	3 (2.36)	7.1e-05	-2.55 (-3.81,-1.29)
Job	0				
6 N(%)		261 (43.43)	77 (60.63)		
5 N(%)		94 (15.64)	20 (15.75)	0.2402	-0.33 (-0.87,0.22)
4 N(%)		114 (18.97)	18 (14.17)	0.0282	-0.63 (-1.18,-0.07)
3 N(%)		79 (13.14)	7 (5.51)	0.0038	-1.2 (-2.02,-0.39)
2 N(%)		31 (5.16)	5 (3.94)	0.2263	-0.6 (-1.58,0.37)
1 N(%)		22 (3.66)	0 (0)	0.9761	-15.35 (-1018.03,987.34)
Height ($\overline{x}, s$)	1	1.62 (0.08)	1.59 (0.09)	3.2e-05	-5.05 (-7.43,-2.67)
PhysicalActivity	0				
None N(%)		166 (27.62)	36 (28.35)		
1h-2h N(%)		116 (19.3)	29 (22.83)	0.61	0.14 (-0.4,0.69)
3h-4h N(%)		105 (17.47)	19 (14.96)	0.56	-0.18 (-0.79,0.43)
>5 N(%)		214 (35.61)	43 (33.86)	0.76	-0.08 (-0.56,0.41)
Reading	0				
Never N(%)		163 (27.12)	70 (55.12)		
Sometimes N(%)		174 (28.95)	31 (24.41)	2.7e-04	-0.88 (-1.35,-0.41)

Table S1: Table with univariate logistic regression analysis for all the variables in the data set.

**variable**	**NA**	**No MCI**	**MCI**	**p-value**	$\hat{\beta}$ **95%CI**($\hat{\beta}$)
Daily N(%)		264 (43.93)	26 (20.47)	4.0e-09	-1.47 (-1.96,-0.98)
OvernightSleepingTime $(\overline{x}, s)$	0	7.07 (1.57)	7.72 (1.83)	6.1e-05	0.24 (0.12,0.36)
DayTimeNap $(\overline{x}, s)$	0	0.56 (0.8)	0.49 (0.69)	0.41	-0.11 (-0.39,0.16)
SleepingTime $(\overline{x}, s)$	0	7.62 (1.75)	8.21 (1.93)	0.001	0.17 (0.07,0.27)
SleepingTimeCAT	0				
6-8h N(%)		411 (68.39)	66 (51.97)		
<6h N(%)		51 (8.49)	10 (7.87)	5.9e-01	0.2 (-0.53,0.93)
>9h N(%)		139 (23.13)	51 (40.16)	8.8e-05	0.83 (0.41,1.24)
Puzzle	0				
Never N(%)		391 (65.06)	94 (74.02)		
Sometimes N(%)		115 (19.13)	16 (12.6)	0.06	-0.55 (-1.12,0.02)
Daily N(%)		95 (15.81)	17 (13.39)	0.30	-0.3 (-0.86,0.27)
Games	0				
Never N(%)		338 (56.24)	66 (51.97)		
Sometimes N(%)		164 (27.29)	37 (29.13)	0.52	0.14 (-0.3,0.59)
Daily N(%)		99 (16.47)	24 (18.9)	0.41	0.22 (-0.3,0.73)
TV.consumption $(\overline{x}, s)$	0	2.29 (0.71)	2.28 (0.77)	0.95	-0.01 (-0.27,0.26)
Smoking	0				
No N(%)		434 (72.21)	103 (81.1)		
Ex N(%)		130 (21.63)	24 (18.9)	0.31	-0.25 (-0.74,0.23)
Smoker N(%)		37 (6.16)	0 (0)	0.98	-16.13 (-1290.87,1258.62)
Alcohol	0				
No N(%)		332 (55.24)	81 (63.78)		
Weekly N(%)		144 (23.96)	23 (18.11)	0.099	-0.42 (-0.93,0.08)
Daily N(%)		125 (20.8)	23 (18.11)	0.275	-0.28 (-0.79,0.22)
Diabetes	0				
No N(%)		463 (77.04)	98 (77.17)		
Yes N(%)		138 (22.96)	29 (22.83)	0.98	-0.01 (-0.46,0.45)
Hypertension	0				
No N(%)		216 (35.94)	48 (37.8)		
Yes N(%)		385 (64.06)	79 (62.2)	0.69	-0.08 (-0.48,0.32)
Hyperlipidemia	0				
No N(%)		336 (55.91)	69 (54.33)		
Yes N(%)		265 (44.09)	58 (45.67)	0.75	0.06 (-0.32,0.45)
Depression	0				
No N(%)		506 (84.19)	89 (70.08)		
Yes N(%)		95 (15.81)	38 (29.92)	0.00024	0.82 (0.38,1.26)
BZD	2				
No N(%)		398 (66.22)	73 (57.48)		
Yes N(%)		201 (33.44)	54 (42.52)	0.055	0.38 (-0.01,0.77)

Table S1: Table with univariate logistic regression analysis for all the variables in the data set.

**variable**	**NA**	**No MCI**	**MCI**	**p-value**	$\hat{\beta}$ **95%CI**($\hat{\beta}$)
NDrugs $(\overline{x}, s)$	15	4.72 (2.66)	4.81 (2.91)	0.72	0.01 (-0.06,0.08)
Memory.Complaint	0				
No N(%)		372 (61.9)	43 (33.86)		
Yes N(%)		229 (38.1)	84 (66.14)	1.9e-08	1.15 (0.75,1.56)
Alone	22				
Alone N(%)		217 (36.11)	49 (38.58)		
Family N(%)		364 (60.57)	76 (59.84)	0.7	-0.08 (-0.47,0.32)
Weight $(\overline{x}, s)$	1	71.24 (12.38)	70.63 (12.35)	0.62	0 (-0.02,0.01)
BMI $(\overline{x}, s)$	1	27.13 (4.31)	28.07 (4.13)	0.026	0.05 (0.01,0.09)
A 02 A	0				
No N(%)		587 (97.67)	127 (100)		
Yes N(%)		14 (2.33)	0 (0)	0.98	-15.04 (-1271.97,1241.9)
A 02 B	0				
No N(%)		402 (66.89)	85 (66.93)		
Yes N(%)		199 (33.11)	42 (33.07)	0.99	0 (-0.41,0.41)
A 02 B A	0				
No N(%)		592 (98.5)	127 (100)		
Yes N(%)		9 (1.5)	0 (0)	0.98	-14.03 (-964.87,936.82)
A 02 B C	0				
No N(%)		411 (68.39)	85 (66.93)		
Yes N(%)		190 (31.61)	42 (33.07)	0.75	0.07 (-0.34,0.47)
A 03 A	0				
No N(%)		595 (99)	125 (98.43)		
Yes N(%)		6 (1)	2 (1.57)	0.57	0.46 (-1.15,2.07)
A 03 F	0				
No N(%)		592 (98.5)	122 (96.06)		
Yes N(%)		9 (1.5)	5 (3.94)	0.08	0.99 (-0.12,2.1)
A 04 AD	0				
No N(%)		601 (100)	125 (98.43)		
Yes N(%)		0 (0)	2 (1.57)	0.98	16.14 (-1207.26,1239.53)
A 06 A	0				
No N(%)		598 (99.5)	127 (100)		
Yes N(%)		3 (0.5)	0 (0)	0.98	-13.02 (-1011.92,985.88)
A 07 A	0				
No N(%)		600 (99.83)	127 (100)		
Yes N(%)		1 (0.17)	0 (0)	0.98	-12.01 (-1061.4,1037.37)
A 10 A	0				
No N(%)		575 (95.67)	125 (98.43)		
Yes N(%)		26 (4.33)	2 (1.57)	0.16	-1.04 (-2.49,0.41)
A 10 B	0				

Table S1: Table with univariate logistic regression analysis for all the variables in the data set.

variable	NA	No MCI	MCI	p-value	$\hat{\beta}$ 95%CI($\hat{\beta}$)
No N(%)		491 (81.7)	104 (81.89)		
Yes N(%)		110 (18.3)	23 (18.11)	0.96	-0.01 (-0.51,0.48)
A 10 B A	0				
No N(%)		550 (91.51)	114 (89.76)		
Yes N(%)		51 (8.49)	13 (10.24)	0.53	0.21 (-0.43,0.85)
A 10 B B	0				
No N(%)		572 (95.17)	124 (97.64)		
Yes N(%)		29 (4.83)	3 (2.36)	0.23	-0.74 (-1.94,0.46)
A 10 B C	0				
No N(%)		599 (99.67)	126 (99.21)		
Yes N(%)		2 (0.33)	1 (0.79)	0.48	0.87 (-1.54,3.27)
A 10 B D	0				
No N(%)		582 (96.84)	124 (97.64)		
Yes N(%)		19 (3.16)	3 (2.36)	0.63	-0.3 (-1.53,0.93)
A 10 B F	0				
No N(%)		594 (98.84)	126 (99.21)		
Yes N(%)		7 (1.16)	1 (0.79)	0.71	-0.4 (-2.5,1.71)
A 10 B G	0				
No N(%)		600 (99.83)	127 (100)		
Yes N(%)		1 (0.17)	0 (0)	0.98	-12.01 (-1061.4,1037.37)
A 10 B X	0				
No N(%)		580 (96.51)	121 (95.28)		
Yes N(%)		21 (3.49)	6 (4.72)	0.51	0.31 (-0.61,1.24)
C 01 A	0				
No N(%)		577 (96.01)	121 (95.28)		
Yes N(%)		24 (3.99)	6 (4.72)	0.71	0.18 (-0.74,1.09)
C 01 B	0				
No N(%)		592 (98.5)	125 (98.43)		
Yes N(%)		9 (1.5)	2 (1.57)	0.95	0.05 (-1.49,1.6)
C 01 D	0				
No N(%)		585 (97.34)	124 (97.64)		
Yes N(%)		16 (2.66)	3 (2.36)	0.85	-0.12 (-1.37,1.13)
C 01 E	0				
No N(%)		570 (94.84)	119 (93.7)		
Yes N(%)		31 (5.16)	8 (6.3)	0.6	0.21 (-0.59,1.01)
C 02 B	0				
No N(%)		598 (99.5)	127 (100)		
Yes N(%)		3 (0.5)	0 (0)	0.98	-13.02 (-1011.92,985.88)
C 02 C A	0				
No N(%)		580 (96.51)	125 (98.43)		

Table S1: Table with univariate logistic regression analysis for all the variables in the data set.

variable	NA	No MCI	MCI	p-value	$\hat{\beta}$ 95%CI($\hat{\beta}$)
Yes N(%)		21 (3.49)	2 (1.57)	0.27	-0.82 (-2.28,0.65)
C 02 L	0				
No N(%)		600 (99.83)	127 (100)		
Yes N(%)		1 (0.17)	0 (0)	0.98	-12.01 (-1061.4,1037.37)
C 03 A	0				
No N(%)		580 (96.51)	127 (100)		
Yes N(%)		21 (3.49)	0 (0)	0.98	-15.05 (-1041.33,1011.24)
C 03 B	0				
No N(%)		581 (96.67)	121 (95.28)		
Yes N(%)		20 (3.33)	6 (4.72)	0.44	0.36 (-0.57,1.3)
C 03 C	0				
No N(%)		541 (90.02)	119 (93.7)		
Yes N(%)		60 (9.98)	8 (6.3)	0.2	-0.5 (-1.26,0.26)
C 03 D	0				
No N(%)		598 (99.5)	124 (97.64)		
Yes N(%)		3 (0.5)	3 (2.36)	0.056	1.57 (-0.04,3.19)
C 03 E	0				
No N(%)		590 (98.17)	125 (98.43)		
Yes N(%)		11 (1.83)	2 (1.57)	0.84	-0.15 (-1.67,1.37)
C 09 A	0				
No N(%)		543 (90.35)	111 (87.4)		
Yes N(%)		58 (9.65)	16 (12.6)	0.32	0.3 (-0.29,0.89)
C 09 B	0				
No N(%)		589 (98)	125 (98.43)		
Yes N(%)		12 (2)	2 (1.57)	0.75	-0.24 (-1.75,1.27)
C 09 C	0				
No N(%)		491 (81.7)	104 (81.89)		
Yes N(%)		110 (18.3)	23 (18.11)	0.96	-0.01 (-0.51,0.48)
C 09 D	0				
No N(%)		518 (86.19)	111 (87.4)		
Yes N(%)		83 (13.81)	16 (12.6)	0.72	-0.11 (-0.68,0.47)
C 09 X	0				
No N(%)		596 (99.17)	125 (98.43)		
Yes N(%)		5 (0.83)	2 (1.57)	0.44	0.65 (-1.01,2.3)
C 10 A	0				
No N(%)		375 (62.4)	80 (62.99)		
Yes N(%)		226 (37.6)	47 (37.01)	0.9	-0.03 (-0.42,0.37)
C 10 A A	0				
No N(%)		395 (65.72)	85 (66.93)		
Yes N(%)		206 (34.28)	42 (33.07)	0.79	-0.05 (-0.46,0.35)

Table S1: Table with univariate logistic regression analysis for all the variables in the data set.

variable	NA	No MCI	MCI	p-value	$\hat{\beta}$ 95%CI($\hat{\beta}$)
C 10 A B	0				
No N(%)		585 (97.34)	124 (97.64)		
Yes N(%)		16 (2.66)	3 (2.36)	0.85	-0.12 (-1.37,1.13)
C 10 A C	0				
No N(%)		600 (99.83)	125 (98.43)		
Yes N(%)		1 (0.17)	2 (1.57)	0.066	2.26 (-0.15,4.67)
C 10 A X	0				
No N(%)		578 (96.17)	121 (95.28)		
Yes N(%)		23 (3.83)	6 (4.72)	0.64	0.22 (-0.7,1.14)
C 10 B	0				
No N(%)		598 (99.5)	127 (100)		
Yes N(%)		3 (0.5)	0 (0)	0.98	-13.02 (-1011.92,985.88)
G 03 C	0				
No N(%)		598 (99.5)	127 (100)		
Yes N(%)		3 (0.5)	0 (0)	0.98	-13.02 (-1011.92,985.88)
G 03 F	0				
No N(%)		600 (99.83)	127 (100)		
Yes N(%)		1 (0.17)	0 (0)	0.98	-12.01 (-1061.4,1037.37)
G 03 X	0				
No N(%)		599 (99.67)	127 (100)		
Yes N(%)		2 (0.33)	0 (0)	0.98	-13.01 (-1236.41,1210.38)
G 04 B	0				
No N(%)		592 (98.5)	127 (100)		
Yes N(%)		9 (1.5)	0 (0)	0.98	-14.03 (-964.87,936.82)
G 04 C	0				
No N(%)		536 (89.18)	119 (93.7)		
Yes N(%)		65 (10.82)	8 (6.3)	0.13	-0.59 (-1.35,0.17)
G 04 C A	0				
No N(%)		559 (93.01)	121 (95.28)		
Yes N(%)		42 (6.99)	6 (4.72)	0.35	-0.42 (-1.29,0.46)
G 04 C B	0				
No N(%)		586 (97.5)	125 (98.43)		
Yes N(%)		15 (2.5)	2 (1.57)	0.54	-0.47 (-1.96,1.02)
G 04 C X	0				
No N(%)		593 (98.67)	127 (100)		
Yes N(%)		8 (1.33)	0 (0)	0.98	-14.03 (-1022.55,994.5)
L 01 X	0				
No N(%)		599 (99.67)	127 (100)		
Yes N(%)		2 (0.33)	0 (0)	0.98	-13.01 (-1236.41,1210.38)
L 02 A	0				

Table S1: Table with univariate logistic regression analysis for all the variables in the data set.

variable	NA	No MCI	MCI	p-value	$\hat{\beta}$ 95%CI($\hat{\beta}$)
No N(%)		600 (99.83)	127 (100)		
Yes N(%)		1 (0.17)	0 (0)	0.98	-12.01 (-1061.4,1037.37)
L 02 B	0				
No N(%)		593 (98.67)	127 (100)		
Yes N(%)		8 (1.33)	0 (0)	0.98	-14.03 (-1022.55,994.5)
M 01 A	0				
No N(%)		469 (78.04)	114 (89.76)		
Yes N(%)		132 (21.96)	13 (10.24)	0.0034	-0.9 (-1.51,-0.3)
M 01 B	0				
No N(%)		600 (99.83)	127 (100)		
Yes N(%)		1 (0.17)	0 (0)	0.98	-12.01 (-1061.4,1037.37)
N 01 B	0				
No N(%)		600 (99.83)	127 (100)		
Yes N(%)		1 (0.17)	0 (0)	0.98	-12.01 (-1061.4,1037.37)
N 02 A	0				
No N(%)		570 (94.84)	121 (95.28)		
Yes N(%)		31 (5.16)	6 (4.72)	0.84	-0.09 (-0.99,0.8)
N 02 B	0				
No N(%)		519 (86.36)	108 (85.04)		
Yes N(%)		82 (13.64)	19 (14.96)	0.7	0.11 (-0.43,0.65)
N 02 BA	0				
No N(%)		596 (99.17)	126 (99.21)		
Yes N(%)		5 (0.83)	1 (0.79)	0.96	-0.06 (-2.21,2.1)
N 02 B B	0				
No N(%)		589 (98)	122 (96.06)		
Yes N(%)		12 (2)	5 (3.94)	0.2	0.7 (-0.36,1.76)
N 02 B E	0				
No N(%)		536 (89.18)	115 (90.55)		
Yes N(%)		65 (10.82)	12 (9.45)	0.65	-0.15 (-0.8,0.5)
N 02 B G	0				
No N(%)		599 (99.67)	127 (100)		
Yes N(%)		2 (0.33)	0 (0)	0.98	-13.01 (-1236.41,1210.38)
N 02 C	0				
No N(%)		598 (99.5)	127 (100)		
Yes N(%)		3 (0.5)	0 (0)	0.98	-13.02 (-1011.92,985.88)
N 04 A	0				
No N(%)		598 (99.5)	126 (99.21)		
Yes N(%)		3 (0.5)	1 (0.79)	0.69	0.46 (-1.81,2.73)
N 04 B	0				
No N(%)		593 (98.67)	125 (98.43)		

Table S1: Table with univariate logistic regression analysis for all the variables in the data set.

variable	NA	No MCI	MCI	p-value	$\hat{\beta}$ 95%CI($\hat{\beta}$)
Yes N(%)		8 (1.33)	2 (1.57)	0.83	0.17 (-1.39,1.73)
N 05 A	0				
No N(%)		591 (98.34)	125 (98.43)		
Yes N(%)		10 (1.66)	2 (1.57)	0.94	-0.06 (-1.59,1.47)
N 05 B	0				
No N(%)		453 (75.37)	87 (68.5)		
Yes N(%)		148 (24.63)	40 (31.5)	0.11	0.34 (-0.08,0.76)
N 05 C	0				
No N(%)		557 (92.68)	117 (92.13)		
Yes N(%)		44 (7.32)	10 (7.87)	0.83	0.08 (-0.64,0.79)
N 06 A	0				
No N(%)		533 (88.69)	97 (76.38)		
Yes N(%)		68 (11.31)	30 (23.62)	0.00031	0.89 (0.4,1.37)
N 06 A A	0				
No N(%)		595 (99)	125 (98.43)		
Yes N(%)		6 (1)	2 (1.57)	0.57	0.46 (-1.15,2.07)
N 06 A B	0				
No N(%)		557 (92.68)	102 (80.31)		
Yes N(%)		44 (7.32)	25 (19.69)	3.3e-05	1.13 (0.6,1.67)
N 06 A X	0				
No N(%)		580 (96.51)	121 (95.28)		
Yes N(%)		21 (3.49)	6 (4.72)	0.51	0.31 (-0.61,1.24)
N 06 B	0				
No N(%)		589 (98)	114 (89.76)		
Yes N(%)		12 (2)	13 (10.24)	3.1e-05	1.72 (0.91,2.53)
N 06 B X	0				
No N(%)		589 (98)	114 (89.76)		
Yes N(%)		12 (2)	13 (10.24)	3.1e-05	1.72 (0.91,2.53)
N 06 C	0				
No N(%)		598 (99.5)	127 (100)		
Yes N(%)		3 (0.5)	0 (0)	0.98	-13.02 (-1011.92,985.88)
N 06 D	0				
No N(%)		592 (98.5)	120 (94.49)		
Yes N(%)		9 (1.5)	7 (5.51)	0.0089	1.34 (0.34,2.35)
N 06 D A	0				
No N(%)		599 (99.67)	123 (96.85)		
Yes N(%)		2 (0.33)	4 (3.15)	0.009	2.28 (0.57,3.98)
N 06 D X	0				
No N(%)		593 (98.67)	122 (96.06)		
Yes N(%)		8 (1.33)	5 (3.94)	0.055	1.11 (-0.02,2.25)

Table S1: Table with univariate logistic regression analysis for all the variables in the data set.

variable	NA	No MCI	MCI	p-value	$\hat{\beta}$ 95%CI($\hat{\beta}$)
R 03 A	0				
No N(%)		569 (94.68)	122 (96.06)		
Yes N(%)		32 (5.32)	5 (3.94)	0.52	-0.32 (-1.28,0.65)
R 03 B	0				
No N(%)		583 (97)	126 (99.21)		
Yes N(%)		18 (3)	1 (0.79)	0.19	-1.36 (-3.38,0.66)
R 03 B A	0				
No N(%)		598 (99.5)	127 (100)		
Yes N(%)		3 (0.5)	0 (0)	0.98	-13.02 (-1011.92,985.88)
R 03 B B	0				
No N(%)		587 (97.67)	126 (99.21)		
Yes N(%)		14 (2.33)	1 (0.79)	0.29	-1.1 (-3.14,0.94)
R 03 C	0				
No N(%)		598 (99.5)	127 (100)		
Yes N(%)		3 (0.5)	0 (0)	0.98	-13.02 (-1011.92,985.88)
R 03 D	0				
No N(%)		594 (98.84)	127 (100)		
Yes N(%)		7 (1.16)	0 (0)	0.98	-14.02 (-1092.18,1064.13)
R 05 C	0				
No N(%)		596 (99.17)	124 (97.64)		
Yes N(%)		5 (0.83)	3 (2.36)	0.15	1.06 (-0.39,2.5)
R 05 C B	0				
No N(%)		596 (99.17)	124 (97.64)		
Yes N(%)		5 (0.83)	3 (2.36)	0.15	1.06 (-0.39,2.5)
R 05 D	0				
No N(%)		600 (99.83)	126 (99.21)		
Yes N(%)		1 (0.17)	1 (0.79)	0.27	1.56 (-1.22,4.34)
R 05 D A	0				
No N(%)		601 (100)	126 (99.21)		
Yes N(%)		0 (0)	1 (0.79)	0.98	15.13 (-1034.26,1064.52)
A.01	0				
No N(%)		598 (99.5)	126 (99.21)		
Yes N(%)		3 (0.5)	1 (0.79)	0.69	0.46 (-1.81,2.73)
A.02	0				
No N(%)		386 (64.23)	84 (66.14)		
Yes N(%)		215 (35.77)	43 (33.86)	0.68	-0.08 (-0.49,0.32)
A.03	0				
No N(%)		586 (97.5)	120 (94.49)		
Yes N(%)		15 (2.5)	7 (5.51)	0.079	0.82 (-0.09,1.74)
A.04	0				

Table S1: Table with univariate logistic regression analysis for all the variables in the data set.

variable	NA	No MCI	MCI	p-value	$\hat{\beta}$ 95%CI($\hat{\beta}$)
No N(%)		601 (100)	125 (98.43)		
Yes N(%)		0 (0)	2 (1.57)	0.98	16.14 (-1207.26,1239.53)
A.06	0				
No N(%)		598 (99.5)	127 (100)		
Yes N(%)		3 (0.5)	0 (0)	0.98	-13.02 (-1011.92,985.88)
A.06A	0				
No N(%)		569 (94.68)	121 (95.28)		
Yes N(%)		32 (5.32)	6 (4.72)	0.78	-0.13 (-1.02,0.77)
A.07	0				
No N(%)		600 (99.83)	127 (100)		
Yes N(%)		1 (0.17)	0 (0)	0.98	-12.01 (-1061.4,1037.37)
A.1O	0				
No N(%)		480 (79.87)	103 (81.1)		
Yes N(%)		121 (20.13)	24 (18.9)	0.75	-0.08 (-0.57,0.41)
A.11	0				
No N(%)		581 (96.67)	125 (98.43)		
Yes N(%)		20 (3.33)	2 (1.57)	0.31	-0.77 (-2.23,0.7)
A.12	0				
No N(%)		528 (87.85)	113 (88.98)		
Yes N(%)		73 (12.15)	14 (11.02)	0.72	-0.11 (-0.72,0.5)
A.16	0				
No N(%)		599 (99.67)	127 (100)		
Yes N(%)		2 (0.33)	0 (0)	0.98	-13.01 (-1236.41,1210.38)
B.01	0				
No N(%)		464 (77.2)	97 (76.38)		
Yes N(%)		137 (22.8)	30 (23.62)	0.84	0.05 (-0.41,0.5)
B.02	0				
No N(%)		600 (99.83)	127 (100)		
Yes N(%)		1 (0.17)	0 (0)	0.98	-12.01 (-1061.4,1037.37)
B.03	0				
No N(%)		580 (96.51)	124 (97.64)		
Yes N(%)		21 (3.49)	3 (2.36)	0.52	-0.4 (-1.63,0.82)
B.05	0				
No N(%)		600 (99.83)	127 (100)		
Yes N(%)		1 (0.17)	0 (0)	0.98	-12.01 (-1061.4,1037.37)
C.01	0				
No N(%)		531 (88.35)	109 (85.83)		
Yes N(%)		70 (11.65)	18 (14.17)	0.43	0.23 (-0.33,0.78)
C.02	0				
No N(%)		576 (95.84)	125 (98.43)		

Table S1: Table with univariate logistic regression analysis for all the variables in the data set.

variable	NA	No MCI	MCI	p-value	$\hat{\beta}$ 95%CI($\hat{\beta}$)
Yes N(%)		25 (4.16)	2 (1.57)	0.18	-1 (-2.45,0.46)
C.03	0				
No N(%)		488 (81.2)	110 (86.61)		
Yes N(%)		113 (18.8)	17 (13.39)	0.15	-0.4 (-0.95,0.15)
C.04	0				
No N(%)		579 (96.34)	122 (96.06)		
Yes N(%)		22 (3.66)	5 (3.94)	0.88	0.08 (-0.91,1.07)
C.05	0				
No N(%)		505 (84.03)	104 (81.89)		
Yes N(%)		96 (15.97)	23 (18.11)	0.55	0.15 (-0.35,0.65)
C.07	0				
No N(%)		515 (85.69)	113 (88.98)		
Yes N(%)		86 (14.31)	14 (11.02)	0.33	-0.3 (-0.9,0.3)
C.08	0				
No N(%)		536 (89.18)	106 (83.46)		
Yes N(%)		65 (10.82)	21 (16.54)	0.072	0.49 (-0.04,1.03)
C.09	0				
No N(%)		342 (56.91)	69 (54.33)		
Yes N(%)		259 (43.09)	58 (45.67)	0.6	0.1 (-0.28,0.49)
C.10	0				
No N(%)		368 (61.23)	78 (61.42)		
Yes N(%)		233 (38.77)	49 (38.58)	0.97	-0.01 (-0.4,0.39)
D	0				
No N(%)		586 (97.5)	121 (95.28)		
Yes N(%)		15 (2.5)	6 (4.72)	0.18	0.66 (-0.31,1.63)
G.01	0				
No N(%)		600 (99.83)	127 (100)		
Yes N(%)		1 (0.17)	0 (0)	0.98	-12.01 (-1061.4,1037.37)
G.03	0				
No N(%)		594 (98.84)	127 (100)		
Yes N(%)		7 (1.16)	0 (0)	0.98	-14.02 (-1092.18,1064.13)
G.04	0				
No N(%)		527 (87.69)	119 (93.7)		
Yes N(%)		74 (12.31)	8 (6.3)	0.056	-0.74 (-1.49,0.02)
H	0				
No N(%)		557 (92.68)	122 (96.06)		
Yes N(%)		44 (7.32)	5 (3.94)	0.17	-0.66 (-1.6,0.29)
J	0				
No N(%)		596 (99.17)	127 (100)		
Yes N(%)		5 (0.83)	0 (0)	0.98	-14.02 (-1289.71,1261.67)

Table S1: Table with univariate logistic regression analysis for all the variables in the data set.

variable	NA	No MCI	MCI	p-value	$\hat{\beta}$ 95%CI($\hat{\beta}$)
L.01	0				
No N(%)		599 (99.67)	127 (100)		
Yes N(%)		2 (0.33)	0 (0)	0.98	-13.01 (-1236.41,1210.38)
L.02	0				
No N(%)		592 (98.5)	127 (100)		
Yes N(%)		9 (1.5)	0 (0)	0.98	-14.03 (-964.87,936.82)
L.04	0				
No N(%)		597 (99.33)	126 (99.21)		
Yes N(%)		4 (0.67)	1 (0.79)	0.88	0.17 (-2.03,2.37)
M.01	0				
No N(%)		467 (77.7)	114 (89.76)		
Yes N(%)		134 (22.3)	13 (10.24)	0.0028	-0.92 (-1.53,-0.32)
M.02	0				
No N(%)		586 (97.5)	118 (92.91)		
Yes N(%)		15 (2.5)	9 (7.09)	0.012	1.09 (0.24,1.94)
M.03	0				
No N(%)		598 (99.5)	126 (99.21)		
Yes N(%)		3 (0.5)	1 (0.79)	0.69	0.46 (-1.81,2.73)
M.04	0				
No N(%)		570 (94.84)	124 (97.64)		
Yes N(%)		31 (5.16)	3 (2.36)	0.19	-0.81 (-2.01,0.39)
M.05	0				
No N(%)		535 (89.02)	114 (89.76)		
Yes N(%)		66 (10.98)	13 (10.24)	0.81	-0.08 (-0.71,0.55)
N.01	0				
No N(%)		600 (99.83)	127 (100)		
Yes N(%)		1 (0.17)	0 (0)	0.98	-12.01 (-1061.4,1037.37)
N.02	0				
No N(%)		485 (80.7)	102 (80.31)		
Yes N(%)		116 (19.3)	25 (19.69)	0.92	0.02 (-0.46,0.51)
N.03	0				
No N(%)		576 (95.84)	118 (92.91)		
Yes N(%)		25 (4.16)	9 (7.09)	0.16	0.56 (-0.22,1.35)
N.04	0				
No N(%)		592 (98.5)	124 (97.64)		
Yes N(%)		9 (1.5)	3 (2.36)	0.49	0.46 (-0.86,1.79)
N.05	0				
No N(%)		410 (68.22)	79 (62.2)		
Yes N(%)		191 (31.78)	48 (37.8)	0.19	0.27 (-0.13,0.66)
N.06	0				

Table S1: Table with univariate logistic regression analysis for all the variables in the data set.

variable	NA	No MCI	MCI	p-value	$\hat{\beta}$ 95%CI($\hat{\beta}$)
No N(%)		514 (85.52)	83 (65.35)		
Yes N(%)		87 (14.48)	44 (34.65)	2e-07	1.14 (0.71,1.57)
N.07	0				
No N(%)		583 (97)	122 (96.06)		
Yes N(%)		18 (3)	5 (3.94)	0.58	0.28 (-0.73,1.29)
P.01	0				
No N(%)		600 (99.83)	127 (100)		
Yes N(%)		1 (0.17)	0 (0)	0.98	-12.01 (-1061.4,1037.37)
R.01	0				
No N(%)		596 (99.17)	125 (98.43)		
Yes N(%)		5 (0.83)	2 (1.57)	0.44	0.65 (-1.01,2.3)
R.03	0				
No N(%)		559 (93.01)	122 (96.06)		
Yes N(%)		42 (6.99)	5 (3.94)	0.21	-0.61 (-1.55,0.34)
R.05	0				
No N(%)		594 (98.84)	123 (96.85)		
Yes N(%)		7 (1.16)	4 (3.15)	0.11	1.02 (-0.23,2.26)
R.06	0				
No N(%)		586 (97.5)	127 (100)		
Yes N(%)		15 (2.5)	0 (0)	0.98	-15.04 (-1229.35,1199.28)
S	0				
No N(%)		531 (88.35)	112 (88.19)		
Yes N(%)		70 (11.65)	15 (11.81)	0.96	0.02 (-0.58,0.61)
V	0				
No N(%)		598 (99.5)	126 (99.21)		
Yes N(%)		3 (0.5)	1 (0.79)	0.69	0.46 (-1.81,2.73)
