# Supplementary material for: Decision Tree for Early Detection of Cognitive Impairment by Community Pharmacists
Source: Front Pharmacol. 2018 Oct 29;9:1232. doi: 10.3389/fphar.2018.01232 (PMC6215965; doi:10.3389/fphar.2018.01232)
Supplement: Supplementary file 2 [file Table_2.PDF]

frontiers

Supplementary Material: Decision tree for early detection of cognitive impairment by community pharmacists

DECISION RULES FOR DISCRIMINANT TREE

These are the rules extracted from the decision tree developed about the final nodes. Information at rules identify with "outcome" ("yes" or "no") if the subject should undergo the tests. "Coverage" is the proportion of individuals in our dataset that meets with that rule and "prob" is the probability of obtaining a positive result of mild cognitive impairment (MCI) in the corresponding tests. The aim of the rules is to achieve a higher effective screening of people which could suffer from MCI.

Rule number: 23 outcome=Yes cover=8 (4%) prob=1.00

- Memory.Complaint = No
- Sex = Female
- ReadingDaily = No
- Age >= 79

Rule number: 7 outcome=Yes cover=19 (9%) prob=0.79

- Memory.Complaint = Yes
- SleepingTime >= 9.25 hours

Rule number: 27 outcome=Yes cover=36 (18%) prob=0.42

- Memory.Complaint = Yes
- SleepingTime < 9.25 hours
- ReadingSometimes = No
- ReadingDaily = No

Rule number: 53 outcome=Yes cover=11 (5%) prob=0.35

- Memory.Complaint = Yes
- SleepingTime < 9.25 hours
- ReadingSometimes = No
- ReadingDaily = Yes
- Age >= 79

Rule number: 45 outcome=Yes cover=13 (6%) prob=0.25

- Memory.Complaint = No
- Sex = Female
- ReadingDaily = No
- Age < 79
- ReadingSometimes = No

Rule number: 12 outcome=No cover=30 (15%) prob=0.14

- Memory.Complaint = Yes
- SleepingTime < 9.25 hours
- ReadingSometimes = Yes

Rule number: 44 outcome=No cover=14 (7%) prob=0.08

- Memory.Complaint = No

- Sex = Female
- ReadingDaily = No
- Age < 79
- ReadingSometimes = Yes

Rule number: 10 outcome=No cover=21 (10%) prob=0.08

- Memory.Complaint = No
- Sex = Female
- ReadingDaily = Yes

Rule number: 52 outcome=No cover=15 (7%) prob=0.05

- Memory.Complaint = Yes
- SleepingTime < 9.25 hours
- ReadingSometimes = No
- ReadingDaily = Yes
- Age <79

Rule number: 4 outcome=No cover=37 (18%) prob=0.04

- Memory.Complaint = No
- Sex = Male
